# Comprehensive analysis of high-throughput transcriptomics to distinguish drug-induced liver injury (DILI) phenotypes

**DOI:** 10.1007/s00204-025-04089-x

**Published:** 2025-06-04

**Authors:** Sangyeon Shin, Chanhee Lee, Taesung Park

**Affiliations:** 1 Interdisciplinary Program in Bioinformatics, Seoul National University, Seoul, Republic of Korea; 2 Department of Statistics, Seoul National University, Seoul, Republic of Korea

**Keywords:** DILI, High-throughput transcriptomics, Concentration–response modeling, New approach methodologies

## Abstract

Drug-Induced Liver Injury (DILI) is a major challenge in drug development, occurring due to liver damage caused by the adverse effects of drugs or xenobiotics. High-throughput transcriptomics (HTTr) provides mechanistic insights into drug-induced hepatotoxicity, complementing traditional chemical structure-based methods. To address the challenges posed by DILI, this study aimed to evaluate the suitability of HTTr data for DILI classification and prediction. Initially, we reviewed the current landscape of HTTr-based DILI research, focusing on public datasets, computational tools, and bioinformatics techniques. Building on this foundation, we analyzed HTTr data from the Open TG-GATEs database, which includes primary human hepatocytes treated with 146 drugs at three concentrations. Gene expression data alone had limited ability to classify DILI phenotypes, performing similarly to chemical structure-based models. However, targeted gene sets improved clustering performance, and changes in clustering performance across concentration levels indicated that concentration information influences toxicity analysis. Machine learning models showed that integrating gene expression and chemical structure data enhanced predictive accuracy, emphasizing the need for multi-modal approaches. These findings underscore HTTr as a valuable tool for advancing DILI classification and prediction, contributing to more reliable drug safety assessments.

**Supplementary Information:**

The online version contains supplementary material available at 10.1007/s00204-025-04089-x.

## Introduction

Drug-Induced Liver Injury (DILI) refers to liver damage caused by adverse effects of drugs or xenobiotics. It is a major concern in clinical pharmacology and drug development, accounting for over 50% of acute liver failure (ALF) cases (Ostapowicz et al. 2002). DILI causes hepatotoxicity that leads to 32% of drugs being withdrawn from the market or failing during clinical trials. It is second most common cause of failure in the drug development process (Babai et al. 2021; Chen et al. 2013b; Moosa et al. 2021; Watkins 2011; Xu et al. 2004). The U.S. Food and Drug Administration (FDA) has identified around 750 drugs that pose a DILI risk, highlighting the widespread nature of this issue (Thakkar et al. 2018). Early and accurate prediction of DILI is crucial not only for improving drug safety but also for reducing financial and time losses associated with late-stage drug failures (Choi et al. 2023). By identifying hepatotoxic candidates in the early phases of development, predictive models can help filter out high-risk compounds, minimizing costly clinical trial failures. Furthermore, improving DILI prediction can prevent severe post-market adverse effects, ultimately protecting patients from life-threatening liver toxicity and reducing the burden on healthcare systems (Raschi and De Ponti 2017).

To predict DILI effectively, various approaches have been developed, including in vivo animal models, in vitro assays, and in silico computational models (Chen et al. 2013a; Wang et al. 2019b; Xu et al. 2008). Animal models have traditionally been used for toxicity prediction. However, their predictive power is limited due to species differences in drug metabolism. In vitro cell-based assays help address some of these limitations by offering human-relevant models, but they often fail to fully capture the systemic effects of drugs. In recent years, to minimize animal testing and enhance predictive accuracy, in silico models have gained attention (Thomas et al. 2019). Among these, Quantitative Structure–Activity Relationship (QSAR) modeling is the commonly used approach due to its cost-effectiveness and efficiency in early hepatotoxicity screening.

QSAR modeling predicts DILI by establishing mathematical relationships between chemical structures and biological activities. By utilizing molecular descriptors such as chemical fingerprints and steric properties, QSAR offers a rapid and resource-efficient approach for identifying potential liver toxicity (Liao et al. 2023). However, QSAR modeling has two main challenges in predicting DILI. First, DILI involves complex, multistep biological processes influenced by diverse chemical agents, and QSAR models primarily relying on chemical structures struggle to capture this complexity. As a result, it becomes difficult to account for the diverse mechanisms of action (MoA) underlying DILI (Matthews et al. 2009). Second, QSAR models do not adequately reflect the complex biological context, including human-specific drug metabolism and pharmacokinetics. Key biological factors like species-specific enzyme activity, transporter variability, and lack of biological context lead to inaccuracies in human predictions (Danishuddin et al. 2021). Thus, while QSAR provides valuable preliminary insights, it lacks sufficient biological depth for a comprehensive understanding of DILI mechanisms. To address these challenges, integrating QSAR with other data-driven approaches, such as transcriptomics and in vitro assays, has been proposed to enhance accuracy and provide deeper insights into DILI mechanisms (Adeluwa et al. 2021; Liao et al. 2023).

High-Throughput Transcriptomics (HTTr) offers promising solutions to these two key challenges of DILI prediction. First, unlike chemical structure analysis, which often lacks transparency (‘black box’ approach) (Shin et al. 2023), HTTr analysis provides comprehensive insights into MoA by profiling global cellular responses across the transcriptome. By revealing detailed molecular-level mechanisms, HTTr not only enhances overall DILI prediction but also allows for improved characterization and prediction of specific DILI phenotypes.

This increased mechanistic clarity, which QSAR modeling generally lacks, is essential for identifying distinct biological pathways associated with different types of liver injury, thereby enabling more precise and informed drug development strategies. Second, HTTr analysis addresses the limitations of animal models in toxicity testing by directly utilizing human cell lines, primary cells, and advanced in vitro systems that better reflect human-specific responses. This approach reduces inaccuracies due to species differences, aligning with regulatory goals emphasizing efficiency and ethical standards in toxicity assessments (ECHA 2016; EPA 2018; Thomas et al. 2019).

In the present study, we aim to determine whether analysis using HTTr data is suitable for classifying and predicting DILI phenotypes. First, we described the existing process of analyzing DILI using HTTr datasets, encompassing materials, methods, and in silico approaches including Biological Pathway Altering Concentration (BPAC) analysis, MoAs studies, and AI-based prediction methods. Next, we analyzed concentration–response gene expression data obtained from the Open TG-GATEs database (Igarashi et al. 2015) as a case study. This dataset is well suited for concentration-based analysis as it provides systematically organized transcriptomics data across multiple dose levels and utilizes primary human hepatocytes, enabling observation of human-relevant toxicological responses. To determine whether DILIConcern of DILIrank (Chen et al. 2016), related to DILI phenotypes, can be classified from a biological perspective, we conducted DEG analysis and a pathway enrichment test to define gene sets and evaluated their clustering ability. Additionally, we calculated BMD to assess the correlation between concentration and gene sets, examining whether genomic data can be valuable for toxicity assessment based on concentration. Furthermore, we confirmed that integrating gene expression data with chemical structure data to train predictive models can enhance prediction performance. In the discussion part, we discussed the importance of HTTr analysis in DILI research and its future directions.

## Materials and methods

### In vitro preclinical models for DILI research

**Fig. 1:**
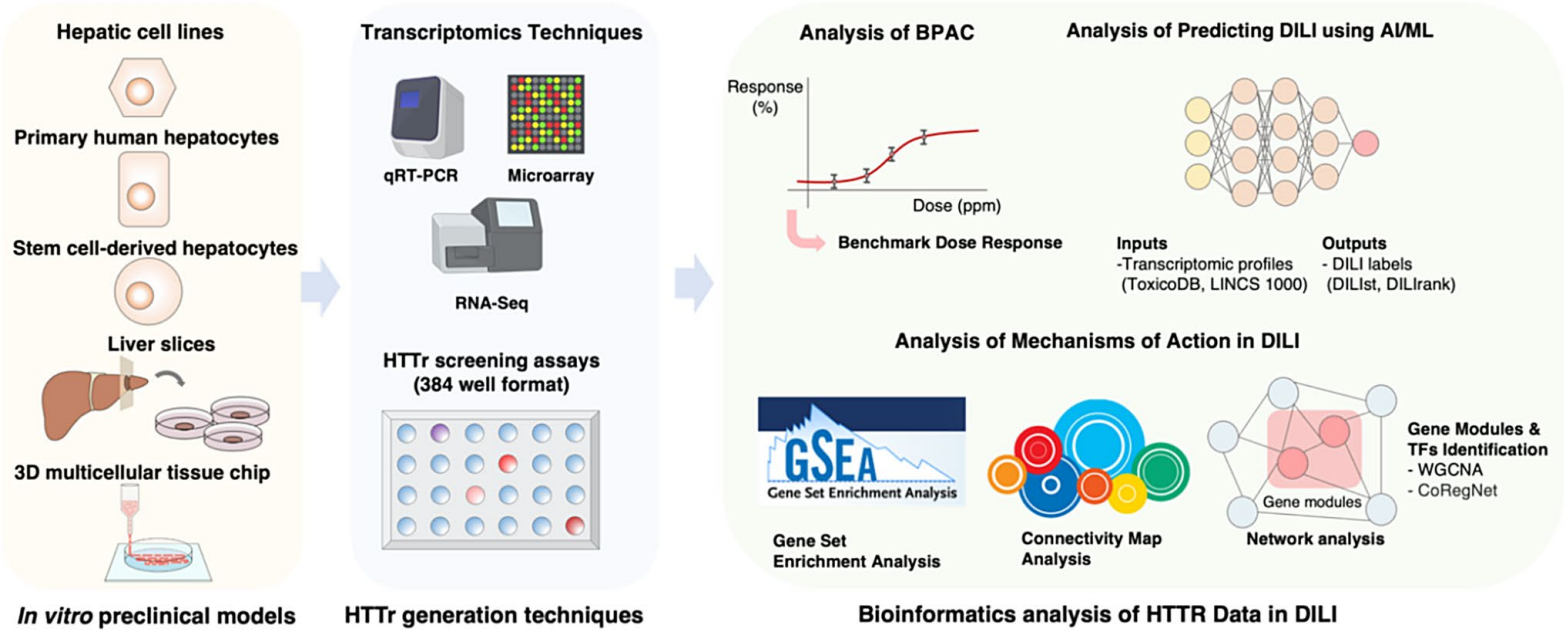
Comprehensive overview of HTTr analysis for DILI in the scope of bioinformatics

Due to DILI’s multifactorial nature, no single in vitro model can fully capture its entire pathophysiological process. A tiered approach incorporating multiple preclinical models has been proposed instead of relying on single testing models (Weaver et al. 2020). Each preclinical model targets specific toxicological endpoints suited to each respective in vitro model (Fig. 1). A three-tiered testing strategy progresses from simple 2D single-cell models (Tier 1) to complex 3D multi-cell systems (Tier 2) and finally incorporates human-specific factors, such as genetics and disease-related elements (Tier 3). In DILI studies, researchers can employ a series of preclinical models designed to characterize hepatotoxicity with increasing precision.

To implement this tiered approach effectively, researchers utilize a range of in vitro models, each offering distinct advantages depending on the level of complexity and biological relevance required. HepG2 cell lines are suitable for basic toxicity assessments, while HepaRG cells and primary human hepatocytes offer more advanced evaluation. HepaRG cells and primary human hepatocytes provide a richer metabolic context and more accurately reflect drug metabolism, enhancing the predictive reliability of hepatotoxicity studies. Stem cell-derived hepatocytes enable modeling individual-specific responses, such as immune-mediated DILI. Liver slices, advanced 2D and 3D tissue chips, and microfluidic systems further enhance the ability to simulate complex drug interactions and toxicity profiles. This tiered approach emphasizes the need to evaluate the suitability of each in vitro test system before conducting extensive HTTr screening. Preclinical models for predicting DILI should align with study objectives, target specific biological pathways, and effectively assess designated chemical groups (Harrill et al. 2019).

### High-throughput transcriptomic techniques

Transcriptomic techniques are categorized by the number of genes profiled and the precision of expression quantification, supporting various applications in toxicology and pharmacology (Fig. 1). qRT-PCR arrays offer high sensitivity and specificity, enabling precise quantification of a limited number of genes. They are ideal for targeted analyses, including pathway interrogation and hypothesis-driven studies (Ates et al. 2018; Sawada et al. 2006). L1000 and S1500 + panels support mid-throughput transcriptomic profiling by measuring the expression of 1000–1500 representative genes. These genes serve as surrogate markers for critical pathways, capturing broader transcriptional activity. This approach offers a balance between coverage and cost-efficiency, facilitating large-scale DILI assessments (Mav et al. 2018; Subramanian et al. 2017). Microarrays and RNA-Seq enable comprehensive transcriptomic profiling. Microarrays support high-throughput gene expression analysis across thousands of genes, while RNA-Seq offers deeper coverage and higher sensitivity, capturing the entire transcriptome, including novel and low-abundance transcripts (Kang et al. 2020; Nair et al. 2020; Rueda-Zárate et al. 2017).

Researchers focusing on specific toxicities, such as genotoxicity or phospholipidosis, might find specialized transcriptomic panels focusing on relevant genes more beneficial. On the other hand, when the toxicity mechanism or affected pathways of a chemical are not well established, a thorough transcriptome analysis could offer a more insightful approach (Harrill et al. 2019).

### In silico analysis of HTTr data in DILI research

HTTr transcends mere data collection; it facilitates the derivation of meaningful interpretations. Through its concentration–response model, HTTr determines potency levels, pinpointing the chemical concentrations that lead to cellular changes. Beyond offering quantitative insights into potential bioactivity thresholds, HTTr data can also be instrumental in hypothesizing the putative mechanisms of action related to chemical toxicity or predicting toxicity of a drug directly. Here, we intend to present a review on toxicity assessment and DILI prediction analysis methods based on HTTr, including biologically pathway altering concentrations (BPACs) analysis, MoA study, and DILI prediction using AI and machine learning.

### Analysis of biological pathway altering concentrations

The In Vitro to In Vivo Extrapolation (IVIVE) framework in HTTr predicts substance behavior in humans using in vitro data. It applies high-throughput toxicokinetic modeling to calculate administered dose equivalents (ADEs) from BPACs, facilitating risk assessment (Harrill et al. 2019). BPAC determination involves selecting chemical data, normalizing transcriptomic datasets, identifying concentration-responsive genes (CRGs) using statistical tests, mapping CRGs to pathways, and estimating pathway potency to determine toxicological effects. Benchmark dose (BMD) modeling, often conducted using BMDExpress software, integrates transcriptomic data to derive dose–response relationships, calculate BMD and its confidence interval, and assess chemical potency for pathway disruption (Phillips et al. 2019; Program 2018). In risk assessment, causal pathway BMDs are used for chemicals with known mechanisms, while the most sensitive pathway BMDs serve as conservative potency markers for data-poor chemicals (Farmahin et al. 2017; Mezencev and Auerbach 2020; Thomas et al. 2013; Webster et al. 2015).

### Analysis of mechanisms of action in DILI

HTTr enables the comparison of gene expression profiles between control and treated groups, providing insights into perturbed biological mechanisms and allowing for the tracking of a drug’s MoAs (Thomas et al. 2019). Differentially expressed gene (DEG) analysis compares treated and control samples to identify significant gene expression changes. Tools, such as DESeq2 (Love et al. 2014), EdgeR (Robinson et al. 2010), and EBSeq (Leng et al. 2013), are used to investigate molecular mechanisms and cellular responses to toxicants (Reiner et al. 2003; Wang et al. 2010). The Connectivity Map (CMap) analyzes gene expression profiles to identify toxicity mechanisms and shared biological targets. It validates chemical relevance and reveals connections between chemicals and biological systems (De Abrew et al. 2019; Harrill et al. 2019). HTTr data are crucial in DILI research for identifying mechanisms of action, utilizing CRG mapping, DEGs analysis, and connectivity mapping, with methods like Gene Set Enrichment Analysis (GSEA) offering sensitivity to subtle genomic variations (Harrill et al. 2019; Subramanian et al. 2005).

### DILI prediction using AI and machine learning approaches

AI techniques are increasingly employed in HTTr analysis to predict DILI occurrence caused by chemical compounds. Machine learning techniques in DILI prediction allow researchers to model complex patterns and interactions by applying computational algorithms and statistical models to analyze large toxicological datasets. Deep learning models have shown superior predictive capabilities over traditional machine learning methods, achieving 97.1% accuracy on the Open TG-GATEs dataset (Feng et al. 2019; Igarashi et al. 2015). Integrating chemical structure data with gene expression profiles and biological knowledge, such as GO enrichment vectors, improves predictive performance and underscores the importance of incorporating biological mechanisms in in silico analyses (Wang et al. 2019a).

### Public datasets for DILI research

To improve the prediction performance of the model, it is crucial to select data that align with the predictive model and research objectives by considering the samples used for data generation, the number of samples, and the production techniques. We investigated publicly available data relevant to HTTr analysis, including analysis platforms, related DBs, preclinical models, sample numbers, and compounds, as shown in Table 1. Three major transcriptomic databases offer expansive resources for research. CMap 1 includes 6100 differential expression profiles from 1,309 chemicals tested on five cell types (Lamb et al. 2006). Its advanced version, LINCS L1000 (CMap 2), features 591,697 profiles from 29,668 perturbations across 98 cell types, estimating 11,350 genes from 978 ‘landmark’ genes using Luminex bead arrays (Lim and Pavlidis 2021; Subramanian et al. 2017). The Open TG-GATEs database focuses on 170 compounds with in vivo (rat) and in vitro (rat and human hepatocyte) transcriptomic data for drug safety evaluations, utilizing microarray technology (Igarashi et al. 2015). In our analysis, we used the Open TG-GATEs dataset, considering primary human hepatocytes to be suitable for DILI research.

**Table 1:** Publicly available HTTr datasets for DILI research

Paper	Analysis platform	Related DB	Preclinical models	Number of samples	Compounds
Lamb et al 2006	Microarray	Cmap	5 types of cell lines	6100 differential expression profiles	1309
Igarashi et al., 2015	Microarray	TG-GATEs	Primary Human Hepatocyte Rat (in vivo, in vitro)	2500 (in vitro) 600 (rat, in vivo)	170
Subramanian et al. 2017	L1000 assays	LINCS L1000	Human Hepatocellular carcinoma	5600 (hepatocytes) 70,000 (all types of cell lines)	240
Shinozawa et al. 2021	scRNA-seq	GSE141183	Human Liver Organoid	5119 cells	238
Koido et al. 2020	AmpliSeq (NGS)	GSE152447	Primary Human Hepatocyte Human Liver Organoid	21 donors 5 donors	12
Podtelezhnikov et al. 2020	RNA-seq	GSE144219	Rat (in vivo)	743	95
Zhang et al. 2023	scRNA-seq	GSE188541	Human Liver Organoid	4600–25,000 cells	4

In addition to the large-scale data produced through the project, we also examined publicly accessible datasets from individual research studies. We collected datasets from the GEO repository by searching for “DILI” and selecting those that included more than one compound. In this case, it is possible to utilize data generated from platforms such as scRNA-seq and RNA-seq, which are less commonly found in large-scale datasets. This approach allows for the selection of data that best fit the research objectives. GES141183 contains scRNA-seq data from 5119 human liver organoid cells treated with 238 compounds at four concentrations. Preprocessing was performed using Seurat v3, with alignment based on the Hg19 genome (Shinozawa et al. 2021). GSE152447 was generated by AmpliSeq to hepatocytes and organoids from 26 donors, testing 12 drugs in three doses for 24–72 h, normalizing to RPM and aligning to Hg19 (Koido et al. 2020). GSE144219 includes RNA-seq data from 743 rat liver samples treated with 95 drugs at single doses. FPKM normalization and OmicSoft Array Studio were used for analysis (Podtelezhnikov et al. 2020). GSE188541 has scRNA-seq data from 4600 to 25,000 organoid cells exposed to four compounds, with Seurat v3 and DESeq2 used for alignment and differential analysis (Zhang et al. 2023).

### Software

Various advanced computer-based toxicity evaluation tools have been developed, as shown in Table 2. BMDExpress2 integrate BMD methods with gene ontology classification to analyze dose–response microarray data, estimating BMD and categorizing genes into biological processes (Phillips et al. 2019; Yang et al. 2007). The tcpl (ToxCast Pipeline) suite supports HTS assay analysis with tools for data storage, normalization, and dose–response modeling, while Tcplfit2 extends these capabilities with advanced curve fitting and BMD modeling for transcriptomic studies (Filer et al. 2016; Sheffield et al. 2022). DILIsym evaluates DILI mechanisms like oxidative stress, mitochondrial dysfunction, and bile acid accumulation by simulating drug impacts on specific pathways (Watkins 2020). ToxSTAR predicts four DILI subtypes (cholestasis, cirrhosis, hepatitis, and steatosis) using machine learning on SMILES input (Shin et al. 2022). VEGAHub provides QSAR models for human toxicology endpoints (Benfenati et al. 2013), while ProTox-II predicts toxicity endpoints like acute toxicity using machine learning on 2D chemical structures (Banerjee et al. 2018). Using tools tailored to multiple data sources could lead to more efficient evaluations of endpoints and toxicity, contributing to advanced computer-based toxicity assessments.

**Table 2:** Software tools used in DILI research

Software	Data type	Main features	Available at
BMDExpress2	Gene expression	BMD calculation CR curve fitting	https://github.com/auerbachs/BMDExpress-2/releases
tcpl	HTS	Data processing CR curve fitting	https://cran.r-project.org/web/packages/tcpl/index.html (R package)
tcplfit2	Gene expression HTS	BMD modeling CR curve fitting	https://cran.r-project.org/web/packages/tcplfit2/index.html (R package)
DILIsym	Mechanistic data	Integrative modeling of DILI mechanisms	https://www.simulations-plus.com/software/dilisym/
ToxSTAR	Chemical structure	QSAR modeling Prediction 4 subtypes of DILI	https://www.kitox.re.kr/toxstar/
VEGA-QSAR	Chemical structure	QSAR modeling Hepatic steatosis	https://www.vegahub.eu/portfolio-item/vega-qsar/
Protox-II	Chemical structure	QSAR modeling Oxidative stress classification	http://tox.charite.de/protox_II

## Materials and methods for open-source data analysis

### Preparing gene expression and chemical structure data

To obtain concentration–response information and gene expression data, the Open TG-GATEs dataset was utilized. In vitro data from human hepatocytes treated with 146 drugs at three concentration levels were specifically selected. In this study, we only used data from the 24-h time point among the eight available time points to ensure sufficient time for the drug to take effect and to capture changes driven solely by concentration information. When analyzing specific gene sets other than the DEG set, average expression values of two biological replicates were utilized as needed. We utilized the DILI Concern information from the DILIrank dataset (Chen et al. 2016). The DILIrank dataset classifies drugs into four categories derived from FDA evaluations, clinical data, and literature reports. The drugs of dataset were annotated by DILIrank, categorizing 102 of the 146 drugs into DILI Concern levels (Most, Less, Ambiguous, No DILI Concern; Supplementary Table 1) and providing severity scores ranging from 0 to 8. The dataset included 50 drugs classified as Most-DILI-Concern, 35 as Less-DILI-Concern, 12 as Ambiguous-DILI-Concern, and 5 as No-DILI-Concern. Gene-DILI associations were validated using the Comparative Toxicogenomics Database (CTD) to further analyze DEG counts, BMD values, and clustering across gene sets.

To compare QSAR and gene expression analysis, chemical structure data representative of QSAR were prepared. SMILES information for each drug was retrieved from PubChem and converted into Morgan fingerprints consisting of 2,048 binary vectors using the Python package RDKit, version 2024.03.5 (RDKit 2024).

### Gene set construction for DILI concern classification and prediction

To analyze the classification and prediction of DILI Concern from a biological perspective, we defined gene sets by categorizing them based on DEGs and enriched pathways. The R package limma, version 3.60.0 (Ritchie et al. 2015), was employed to identify genes with significant expression changes at different concentrations. Genes with an adjusted *p* value < 0.05 were designated as DEGs, without applying a specific logFC threshold. To identify biological pathways significantly affected by these DEGs, KEGG pathway enrichment analysis was conducted using the Python package gseapy, version 1.1.3 (Fang et al. 2022) with an adjusted *p* value threshold of < 0.05.

### BMD value calculation

BMDExpress2, version 2.3, was used to track concentration-responsive changes and calculate BMD values. In this study, BMD values were derived using DEG counts for each drug across different concentrations as the response variable. The concentrations corresponded to three relative levels (10:30:100) as specified in the Open TG-GATEs dataset. Consequently, the resulting BMD values do not represent absolute concentrations but serve to compare the relative ranking of groups in terms of toxicity-indicating concentrations. Benchmark dose analysis was performed using continuous models (Hill, Power, Linear, Poly2, Exp2, Exp3, Exp4, and Exp5) with a 95% confidence level and a BMR factor of 1 SD. The best polynomial model was selected using a nested Chi-square test with a p-value cutoff of 0.05.

## Results

### Comparing chemical structure data and gene expression data

**Fig. 2:**
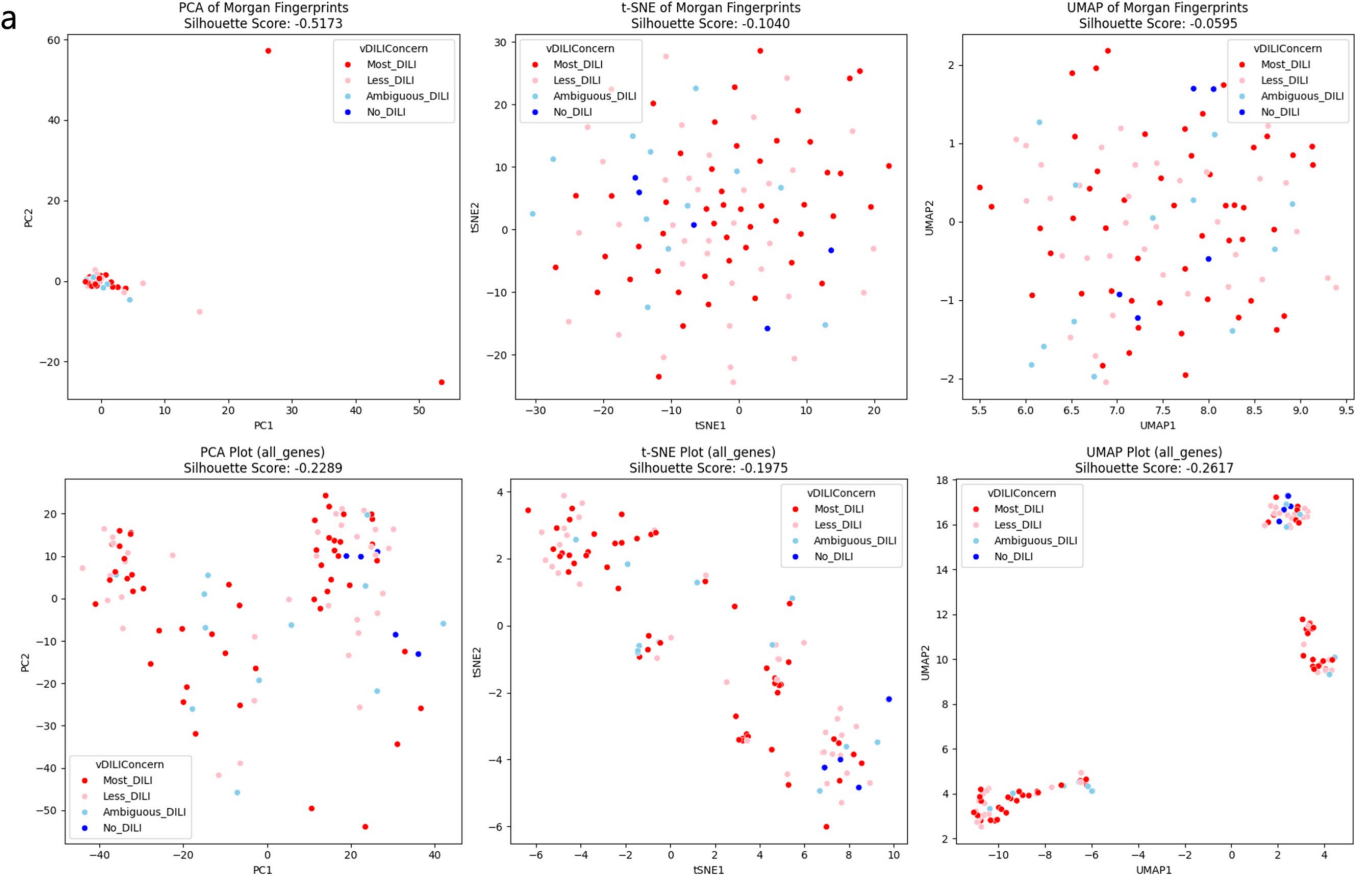
Dimension reduction analysis on chemical structure and gene expression data. To compare the data in a lower-dimensional space PCA, t-SNE, and UMAP were applied to both chemical structure data and gene expression data

To determine whether HTTr is suitable for DILI phenotype analysis, we compared the classification performance of DILI Concern using HTTr data, and chemical structure data, which has traditionally been used for DILI prediction. Dimensionality reduction methods, including Principal Component Analysis (PCA), t-Distributed Stochastic Neighbor Embedding (t-SNE), and Uniform Manifold Approximation and Projection (UMAP), were applied (Fig. 2). Clustering performance was evaluated using the silhouette score, which measures intra-label distance. Silhouette scores for DILI Concern were low in both cases (Gene expression data: − 0.2617, Chemical structure data: − 0.0595). Since silhouette scores need to be 0 or higher to meaningfully discuss clustering quality, it is challenging to conclude that gene expression data are inherently better for distinguishing DILI Concern.

### Different expression patterns between gene sets and concentration levels

**Fig. 3:**
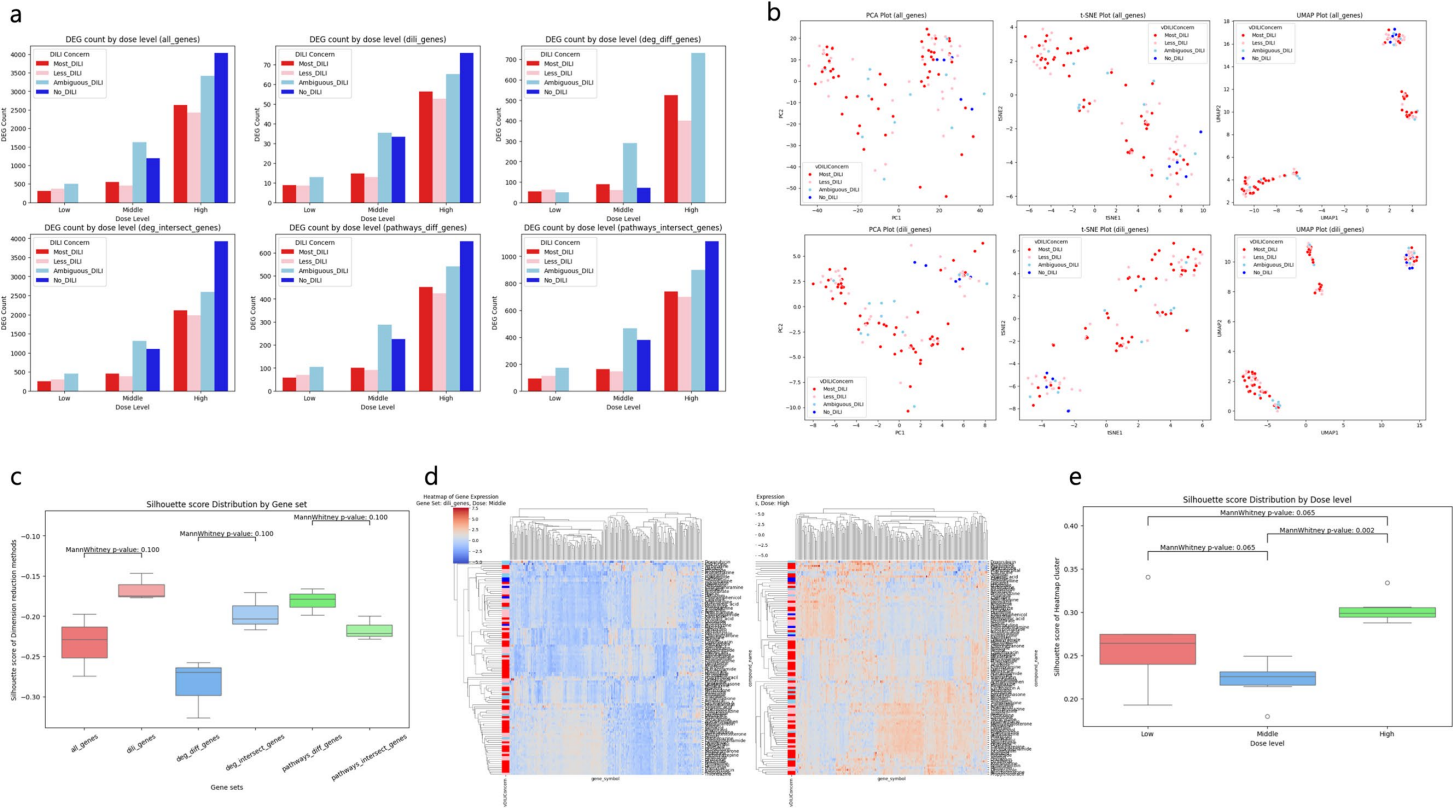
Different expression patterns between Gene sets and Concentration levels. To compare changes in DEG counts across concentrations for different gene sets, a bar plot was used for visualization (**a**). Dimension reduction methods such as PCA, t-SNE, and UMAP were applied to the gene sets for visualization (b, Supplementary Fig. 2). The distribution of silhouette scores from (**b**) was compared using a box plot (**c**). Additionally, gene expression levels across concentrations for each gene set were visualized using heatmaps, and silhouette scores of clusters derived from hierarchical analysis were compared (d, e, Supplementary Fig. 3)

A key strength of gene expression data is its suitability for biological interpretation, which can be enhanced by defining relevant gene sets. To this end, DEG analysis and KEGG pathway enrichment analysis were performed. We defined six gene sets based on the DEG and pathway differences between Most- and No-DILI Concern groups: (1) All genes (*n* = 19,933), (2) DILI-related genes (*n* = 213), (3) genes from the difference set of DEGs between Most- and No-DILI Concern (*n* = 7190), (4) genes from the intersect set of DEGs between Most- and No-DILI Concern (*n* = 10,435), (5) genes from the difference set of pathways between Most- and No-DILI Concern (*n* = 1937), and (6) genes from the intersect set of pathways between Most- and No-DILI Concern (*n* = 3389) (Supplementary Fig. 1; List of genes is in Supplementary Table 1). Analyzing correlations between gene sets and DILI concern labels can reveal biological processes influencing DILI concern levels (Fig. 3). DEG counts were calculated for each gene set across different concentrations. While DEG counts increased with drug concentration, no distinct differences were observed between gene sets (Fig. 3a). However, silhouette score distribution from dimension reduction analysis revealed slight differences in clustering depending on the defined gene set (Fig. 3b, c; Mann–Whitney U test p value: 0.100). In particular, the score difference between the All gene set and the DILI-related gene set suggests that using targeted gene sets related to DILI is more appropriate for analyzing DILI Concern.

Heatmaps of gene expression by concentration and gene set revealed clustering patterns and differentiation based on DILI Concern (Fig. 3d, e). While silhouette scores of clusters formed by heatmaps showed no significant differences between gene sets, clustering was significantly influenced by drug concentration. For example, the silhouette score distribution at high concentration is greater than at moderate level (Fig. 3e; Mann–Whitney *U* test *p* value: 0.002). This result indicates that concentration is a significant factor in heatmap clustering.

These findings highlight the importance of selecting target gene sets based on toxicity-related phenotypes and incorporating concentration information to enable more sensitive observations in toxicity studies based on genomic data. This suggests that HTTr is well suited for addressing the limitations of DILI research, particularly in overcoming the complexity of MoA.

### Comparing BMD values between gene sets and DILI concern levels

**Fig. 4:**
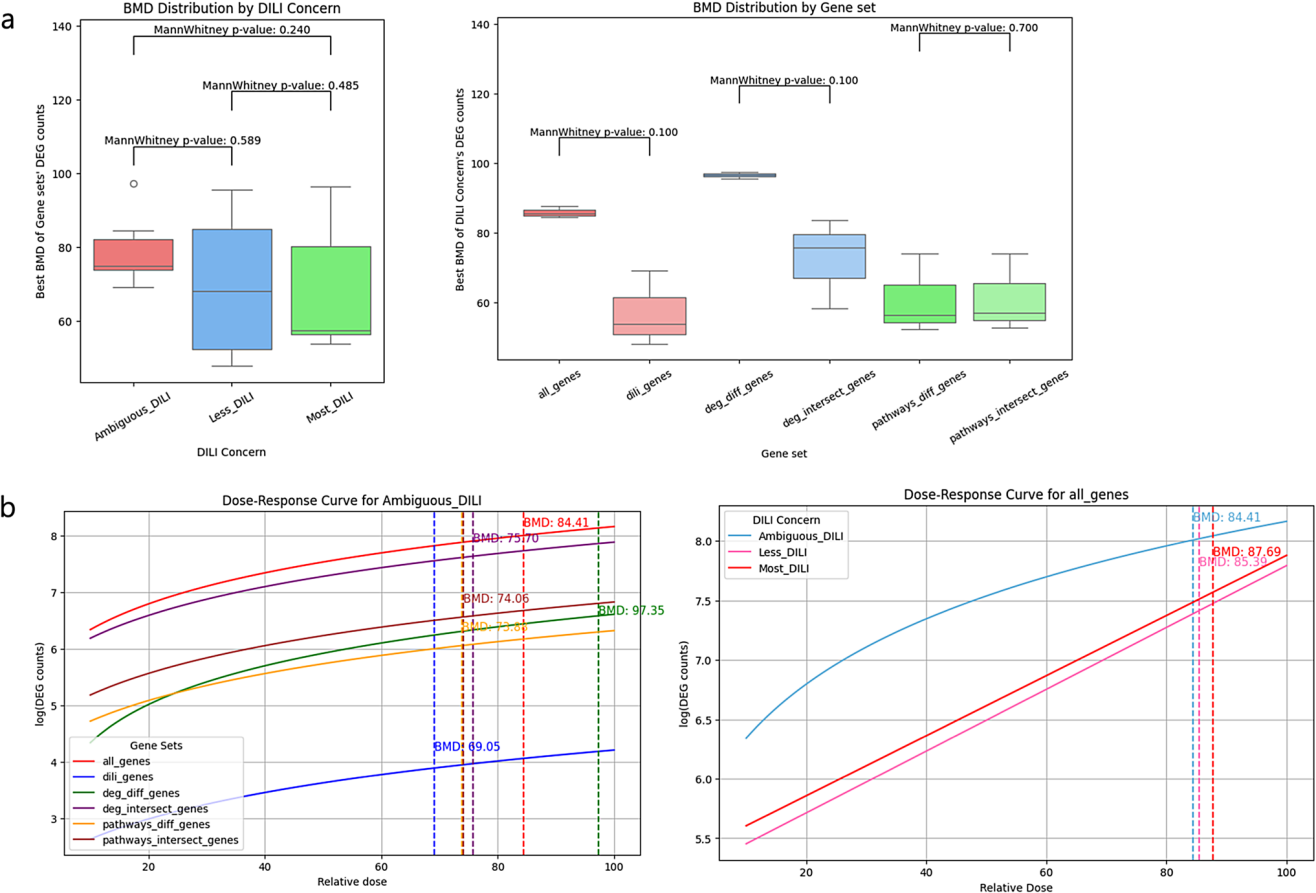
Comparing BMD values between Gene sets and DILI Concern levels. BMD values were calculated using BMDExpress2 based on DILI Concern levels and gene sets, and their distributions were compared using box plots (**a**). For the cases in (**a**), fitted curves from BMDExpress2 were visualized, with the corresponding BMD values indicated as dashed lines for comparison (**b**)

In toxicology, analyzing concentration is crucial for identifying toxic doses and monitoring biological process changes. This also suggests that HTTr can help overcome the limitation of DILI research, where experimental results from animal models are difficult to translate to humans. Comparing benchmark doses across gene sets or DILI concern groups provides biological insights related to concentration effects. We calculated BMD values for DEG counts across DILI Concern groups. Representing summarized gene expression data instead of individual gene values, this approach enhances robustness by reducing variability and emphasizing overall transcriptional changes (Fig. 4). DEG counts were determined for each drug at different concentrations, and these data points were used for concentration–response curve fitting to calculate BMD values. Curve fitting could not be performed for the No-DILI group due to insufficient concentration data (only two points per drug).

When examining the BMD value distributions for each DILI Concern group across gene sets, the DILI-related gene set showed slightly lower BMD values compared to the All gene set (Fig. 4a; Mann–Whitney U test p value: 0.100, Fig. 4b; BMD value of All gene set: 84.41, DILI-related gene set: 69.05 for Ambiguous DILI). A lower BMD indicates substantial changes at lower concentrations, suggesting that more specific gene sets are associated with higher sensitivity, as they exhibit significant DEG changes at lower concentrations (Fig. 4a, b). However, no significant differences in BMD value distributions were observed between DILI Concern groups for any of the gene sets.

These results suggest that specific gene sets may improve sensitivity to concentration-dependent biological changes. Therefore, using HTTr data allows researchers to define gene sets based on their study objectives, leading to more refined and precise research outcomes. Selection of developed measurements to represent gene expression patterns—beyond DEG counts—could further enhance the detection of biologically meaningful, concentration-driven changes.

### Predicting DILI concern levels using chemical structure and gene expression data

To evaluate the effectiveness of different data types for predicting DILI Concern levels, machine learning models, such as Support Vector Machine (SVM), Random Forest (RF), and Deep Neural Network (DNN), were applied to chemical structure data, gene expression data, and combined datasets. Prediction was performed with fivefold cross validation, and the performance was assessed using accuracy. The results were also obtained using PCA-derived components to account for differences in feature size.

**Fig. 5:**
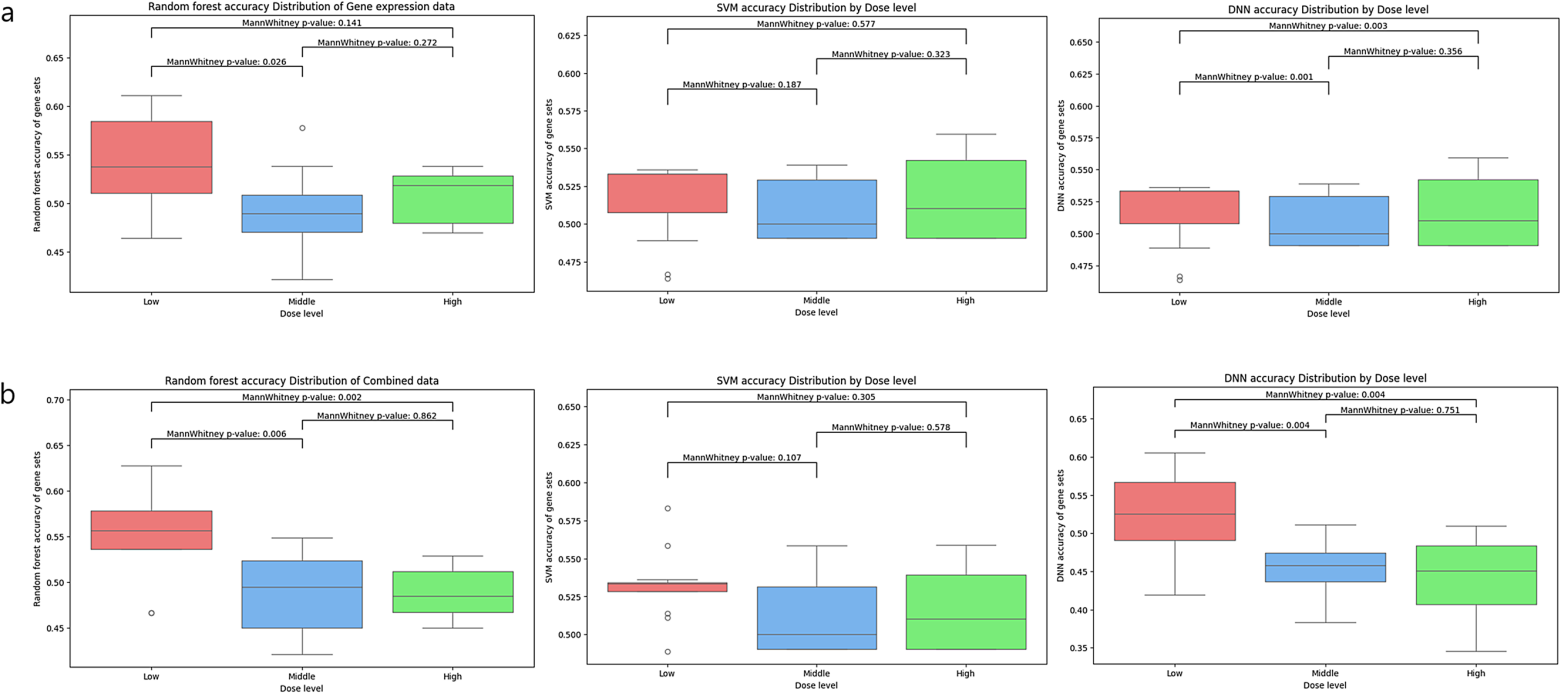
Prediction DILI Concern by SVM, Random Forest (RF) and DNN. To examine differences across concentrations, the distribution of each prediction method accuracy was analyzed for gene expression data (**a**) and combined data (**b**)

Prediction accuracy for gene expression data varied with dose level and gene set selection. Accuracy at lower doses using gene expression data exceeded that of chemical structure data (Table 3), Supplementary Table 2; SVM accuracy of Gene expression data: 0.5333 ± 0.03, Chemical structure data: 0.4705 ± 0.04). The highest accuracy among all cases was achieved when predicting with RF using combined data, low-dose concentration, the pathways difference gene set, and PCA for dimension reduction (Table 4, Supplementary Table 2; RF accuracy: 0.6278 (± 0.11)). When examining the distribution of RF’s prediction accuracy across gene sets at different concentrations, both gene expression data alone and combined data showed significantly higher accuracy at low concentrations compared to middle or high concentrations (Fig. 5a, b). This indicates that prediction performance can vary depending on concentration levels, underscoring the importance of incorporating concentration information when predicting DILI-related phenotypes. These results suggest that integrating multiple data types can enhance the predictive performance of machine learning models for DILI Concern, highlighting the value of combining complementary information sources.

**Table 3:** Prediction accuracy by using gene expression and chemical structure data

Data type	Dose level	SVM	RF	DNN	SVM(PCA)	RF(PCA)	DNN(PCA)
all_genes	Low	**0.5333 (± 0.03)**	**0.5611 (± 0.10)**	**0.5194 (± 0.15)**	**0.5139 (± 0.07)**	**0.5167 (± 0.09)**	**0.5889 (± 0.17)**
all_genes	Middle	0.4905 (± 0.01)	0.4700 (± 0.04)	0.4800 (± 0.10)	0.4639 (± 0.06)	0.4667 (± 0.08)	0.5167 (± 0.15)
all_genes	High	0.4905 (± 0.01)	0.5186 (± 0.06)	0.4614 (± 0.05)	0.4889 (± 0.09)	0.6111 (± 0.14)	0.5361 (± 0.16)
Morgan Fingerprint	–	0.4705 (± 0.04)	0.4805 (± 0.05)	0.4610 (± 0.02)	0.4905 (± 0.01)	0.4314 (± 0.04)	0.4810 (± 0.06)

Models with the best performance are highlighted in bold text

**Table 4:** Prediction accuracy by using combined data

Gene set	Dose level	SVM	RF	DNN	SVM(PCA)	RF(PCA)	DNN(PCA)
all	Low	0.5333 (± 0.03)	0.4667 (± 0.12)	0.5833 (± 0.08)	0.5139 (± 0.07)	0.5389 (± 0.08)	**0.6056 (± 0.11)**
dili	Low	0.5333 (± 0.03)	0.5361 (± 0.06)	**0.6056 (± 0.15)**	0.5111 (± 0.04)	0.5556 (± 0.09)	0.5361 (± 0.16)
deg_diff	Low	0.5333 (± 0.03)	0.5361 (± 0.06)	0.4250 (± 0.14)	0.4889 (± 0.09)	0.4667 (± 0.11)	0.5139 (± 0.10)
deg_intersect	Low	0.5333 (± 0.03)	0.5583 (± 0.14)	0.5611 (± 0.10)	0.5361 (± 0.06)	0.5639 (± 0.12)	0.4917 (± 0.07)
pathways_diff	Low	0.5333 (± 0.03)	0.5806 (± 0.06)	0.4194 (± 0.06)	**0.5833 (± 0.03)**	**0.6278 (± 0.11)**	0.5583 (± 0.13)
pathways_intersect	Low	0.5333 (± 0.03)	**0.5833 (± 0.11)**	0.5111 (± 0.11)	0.5583 (± 0.04)	0.5778 (± 0.11)	0.4889 (± 0.13)

Models with the best performance are highlighted in bold text

## Discussion and conclusion

HTTr has been pivotal in uncovering DILI’s complex molecular dynamics, offering deeper insights into hepatotoxic mechanisms and supporting the transition to NAMs (Harrill et al. 2019; Li et al. 2020; Shinozawa et al. 2021). We reviewed the use of HTTr in DILI research, initially focusing on the tools and methods used in HTTr, like preclinical models, and transcriptomic techniques. Then, we examined entire process of HTTr data analysis in DILI, emphasizing BPAC, MoAs, and AI approaches. Furthermore, we summarized relevant public datasets and software tools, offering essential resources for HTTr research.

We analyzed and predicted the degree of DILI Concern from a genomic perspective using publicly available HTTr data from Open TG-GATEs. This included DEG analysis, pathway enrichment analysis, BMD calculations, and machine learning predictions as a case study. The findings suggest that gene expression data provide greater ability to distinguish DILI-related biological meaning compared to chemical structure data. We compared BMD values and clustering performance across concentrations and gene sets. The results suggest that DILI-related genes exhibit better clustering performance and lower BMD values rather than all genes (Fig. 3, 4), providing insights into their biological significance. Although the prediction accuracy by machine learning and DNN prediction was not high due to the unbalanced dataset, models using gene expression data demonstrated better performance than those relying on chemical structure data (Fig. 5). Moreover, the enhanced predictive performance observed when combining gene expression data with chemical structure data. Using a DNN model with all genes at low concentrations, accuracy increased from 0.5194 with gene expression data alone to 0.5833 with combined data. This highlights the benefits of using gene expression data, including enhanced prediction capabilities, and emphasizes the need to develop future analytical methods that integrate multiple data modalities.

The future of HTTr in DILI research is poised for significant progress, emphasizing the development of innovative techniques and methodologies to improve DILI understanding and prediction. Advancements in deep learning models are expected to leverage vast amounts of transcriptomic data to improve DILI prediction (Li et al. 2020). Additionally, novel HTTr platforms, such as TempO-Seq, are being employed for concentration–response modeling. These platforms address challenges related to throughput and cost, enabling more efficient identification and interpretation of biological-response pathways in DILI (Ramaiahgari et al. 2019). Research into circulating microRNAs in human serum is also expected to yield new biomarker candidates, providing mechanistic insights and aiding in the development of effective diagnostic tools for DILI (Krauskopf et al. 2015). The development of in vitro transcriptomic assays using advanced models, such as HEPATOPAC and human liver organoids, is expected to reduce DILI risk early in drug development. These models offer high sensitivity and specificity for detecting hepatotoxicants and distinguishing drugs with lower DILI risk (Kang et al. 2020). Additionally, network-based transcriptome analysis, such as weighted correlation network analysis (WGCNA) and graph neural network (GNN), is expected to enhance the mechanistic interpretation of toxicogenomic data. This approach can identify candidate determinants of DILI and improve understanding of the molecular mechanisms underlying hepatotoxicity (Wijaya et al. 2023). These future directions in HTTr research for DILI have the potential to transform our ability to understand, predict, and mitigate drug-induced liver injury, ultimately leading to safer pharmaceuticals and personalized medicine strategies.

In conclusion, the advancement of HTTr in DILI research stands to significantly enhance the evaluation of drug safety. Our study outlined the current state of HTTr analysis and discussed future directions. It is imperative for the scientific community to refine HTTr methods further, to better elucidate DILI mechanisms, and to leverage emerging technologies, which include sophisticated preclinical models that mimic human physiology and AI algorithms proficient in handling large-scale datasets. This multifaceted evaluation strategy will improve the precision and reliability of safety assessments. With ongoing technological and methodological advancements and a dedication to rigorous, multi-angled research, the future promises a marked reduction in drug-induced liver injuries. This aligns with the broader goal of optimizing patient outcomes and advancing pharmacology through safer and more effective therapeutic interventions.

## Supplementary Information

Below is the link to the electronic supplementary material.

Supplementary file1 (DOCX 1535 KB)

Supplementary file2 (XLSX 709 KB)

Supplementary file3 (XLSX 17 KB)

## Data Availability

The transcriptomic and chemical data used in this study were obtained from the publicly available Open TG-GATEs dataset. The dataset was downloaded via the ToxicoDB platform (https://www.toxicodb.ca/datasets/1). All analyses were performed gene expression profiles provided in this dataset.
