# Supplementary material for: Comprehensive analysis of high-throughput transcriptomics to distinguish drug-induced liver injury (DILI) phenotypes
Source: Arch Toxicol. 2025 Jun 4;99(9):3721–34. doi: 10.1007/s00204-025-04089-x (PMC12408718; doi:10.1007/s00204-025-04089-x)
Supplement: Supplementary file 1 — Supplementary file1 (DOCX 1535 KB) [file 204_2025_4089_MOESM1_ESM.docx]

**­­­­Comprehensive analysis of high-throughput transcriptomics to distinguish drug-induced liver injury (DILI) phenotypes**

Sangyeon Shin^1^, Chanhee Lee^1^, Taesung Park^1,2^

^1^Interdisciplinary Program in Bioinformatics, Seoul National University, Seoul, Republic of Korea.

^2^Department of Statistics, Seoul National University, Seoul, Republic of Korea.
Email: [tspark@stats.snu.ac.kr](mailto:tspark@stats.snu.ac.kr).

**Contributions**

Sangyeon Shin and Chanhee Lee contributed equally to this work.

**Corresponding author**

Correspondence to Taesung Park

**Conflict of interest**

The authors have no competing interests to declare that are relevant to the content of this article.

**Funding**

This work was supported by the Korea Institute of Toxicology (KIT) Research Program (No. 1711195881).

**Supplementary Figure 1**

**
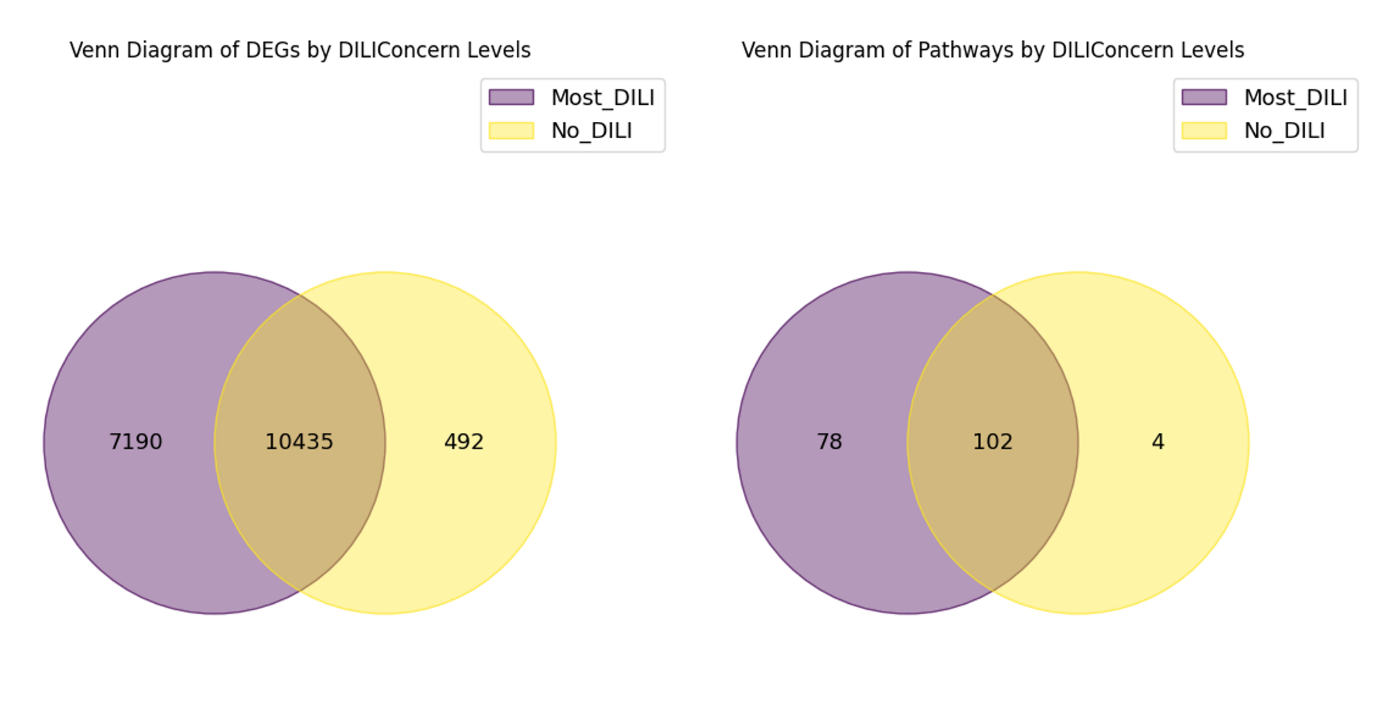
**

Supplementary Figure 1. Detailed criteria and methods for dividing the gene sets

To compare the analysis results across gene sets, we categorized the gene sets into various groups. DEG analysis and pathway enrichment analysis were conducted, and Venn diagrams were used to determine the difference sets and intersection sets of DEGs (left) and enriched pathways (right) between drugs classified as Most-DILI and No-DILI Concern. Based on this, six gene sets were defined as follows: (1) All genes covered in the Open TG-GATEs dataset, (2) Genes defined as DILI-related according to data obtained from CTD, and (3-6) Genes could be categorized based on the Venn diagram.

**Supplementary Figure 2**

**
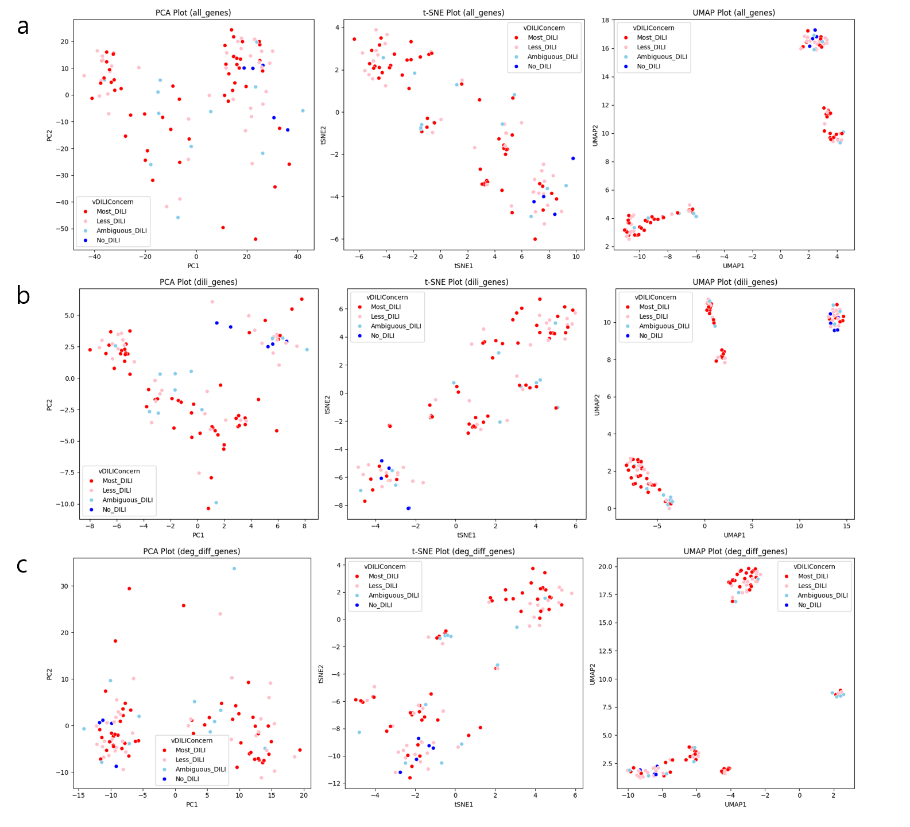

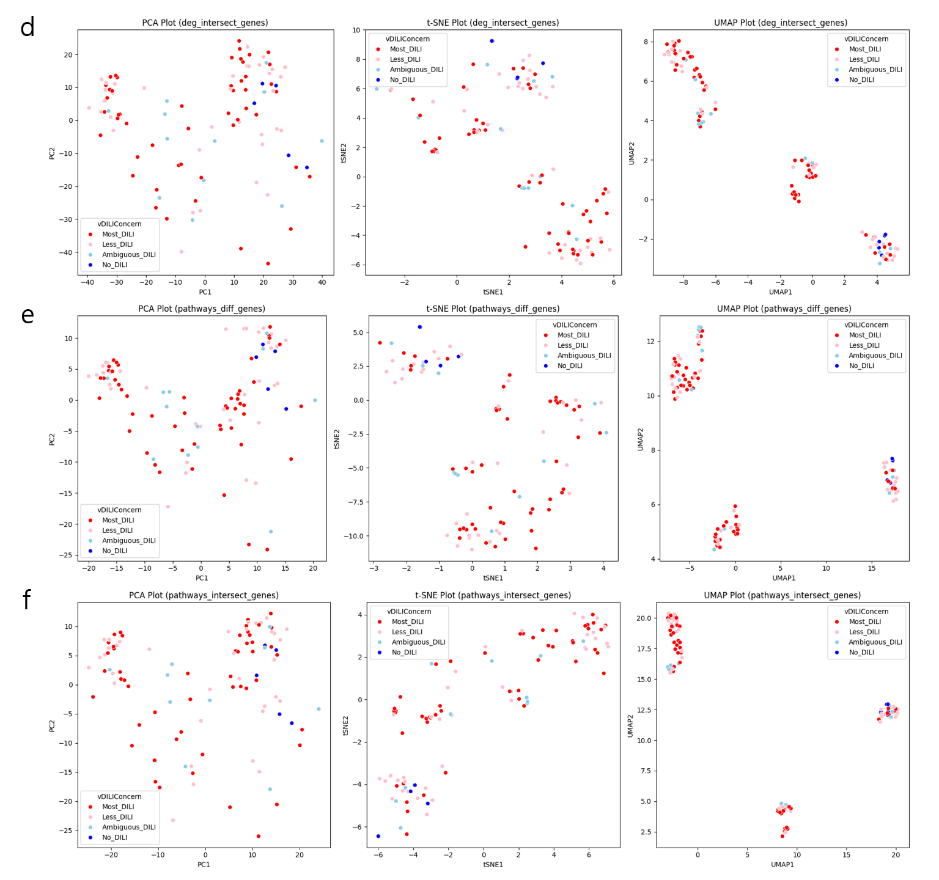
**

**Supplementary Figure 3**

**
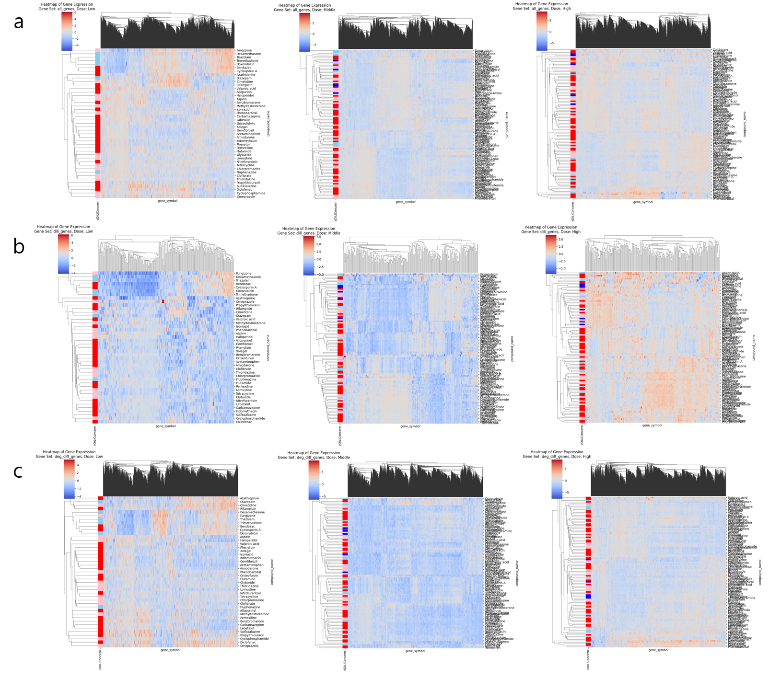

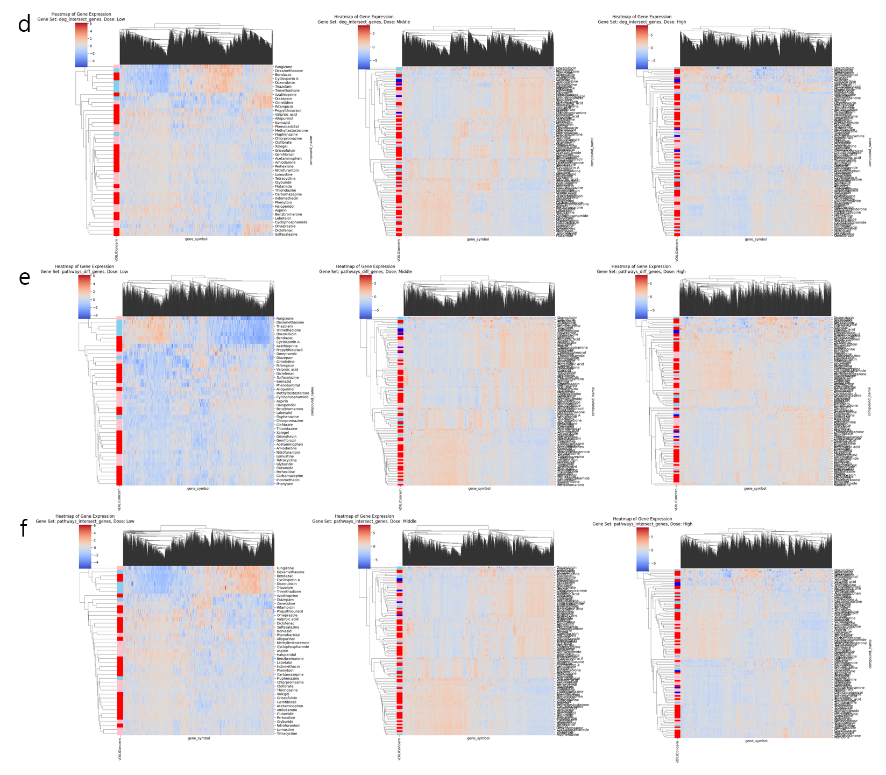
**
